# Breast cancer differential diagnosis using diffuse optical spectroscopic imaging and regression with *z*-score normalized data

**DOI:** 10.1117/1.JBO.26.2.026004

**Published:** 2021-02-23

**Authors:** Jeffrey M. Cochran, Anais Leproux, David R. Busch, Thomas D. O’Sullivan, Wei Yang, Rita S. Mehta, Alice M. Police, Bruce J. Tromberg, Arjun G. Yodh

**Affiliations:** a University of Pennsylvania, Department of Physics and Astronomy, Philadelphia, Pennsylvania, United States; b University of California Irvine, Beckman Laser Institute and Medical Clinic, Irvine, California, United States; c University of Texas Southwestern Medical Center, Departments of Anesthesiology and Pain Management & Neurology and Neurotherapeutics, Dallas, Texas, United States; d University of Notre Dame, Department of Electrical Engineering, Notre Dame, Indiana, United States; e University of Texas MD Anderson Cancer Center, Department of Diagnostic Radiology, Houston, Texas, United States; f University of California Irvine, Department of Medicine, Irvine, California, United States; g Northwell Health Breast Care Centers, Sleepy Hollow, New York, United States; h National Institute of Biomedical Imaging and Bioengineering, National Institutes of Health, Bethesda, Maryland, United States

**Keywords:** diffuse optics, differential diagnosis, breast cancer, biopsy

## Abstract

**Significance**: Current imaging paradigms for differential diagnosis of suspicious breast lesions suffer from high false positive rates that force patients to undergo unnecessary biopsies. Diffuse optical spectroscopic imaging (DOSI) noninvasively probes functional hemodynamic and compositional parameters in deep tissue and has been shown to be sensitive to contrast between normal and malignant tissues.

**Aim**: DOSI methods are under investigation as an adjunct to mammography and ultrasound that could reduce false positive rates and unnecessary biopsies, particularly in radiographically dense breasts.

**Methods**: We performed a retrospective analysis of 212 subjects with suspicious breast lesions who underwent DOSI imaging. Physiological tissue parameters were z-score normalized to the patient’s contralateral breast tissue and input to univariate logistic regression models to discriminate between malignant tumors and the surrounding normal tissue. The models were then used to differentiate malignant lesions from benign lesions.

**Results**: Models incorporating several individual hemodynamic parameters were able to accurately distinguish malignant tumors from both the surrounding background tissue and benign lesions with area under the curve (AUC) ≥0.85. Z-score normalization improved the discriminatory ability and calibration of these predictive models relative to unnormalized or ratio-normalized data.

**Conclusions**: Findings from a large subject population study show how DOSI data normalization that accounts for normal tissue heterogeneity and quantitative statistical regression approaches can be combined to improve the ability of DOSI to diagnose malignant lesions. This improved diagnostic accuracy, combined with the modality’s inherent logistical advantages of portability, low cost, and nonionizing radiation, could position DOSI as an effective adjunct modality that could be used to reduce the number of unnecessary invasive biopsies.

## 1 Introduction

In standard clinical practice, breast lesions are imaged using x-ray mammography and ultrasound;^1^ this requires the differentiation of suspicious lesions from surrounding healthy tissue. Additional diagnostic imaging and/or invasive biopsies are then performed to determine whether the suspicious lesion is malignant or benign.^1^ Thereafter, treatment plans for patients with malignant carcinomas are developed. Unfortunately, although x-ray mammography has very high sensitivity to breast tumors, it has relatively low specificity,^2^ which produces a high false positive rate, i.e., ∼10% across all ages,^3^ with higher rates in younger patients.^4^ Moreover, ultrasound imaging is also susceptible to this high false positive rate,^5^ which prompts more than 500,000 unnecessary negative biopsies per year^5^^,^^6^ and can lead to excessive cost^3^^,^^7^ and stress for patients.^8^ Additionally, women with radiographically dense breasts, who may be at increased risk for breast cancer,^9^^,^^10^ are more difficult to image with mammography, leading to even higher false positive rates.^11^ Functional information can help distinguish benign lesions from the more metabolically active malignant tumors. Indeed, more functional information about the lesions could improve the high false positive rate of x-ray mammography. Thus, other imaging modalities such as positron emission tomography (PET) or magnetic resonance imaging (MRI) can augment the diagnostic ability of mammography, albeit with logistical constraints such as ionizing radiation, cost, and low throughput.

Diffuse optical imaging and monitoring technologies hold potential to improve current breast cancer diagnosis paradigms.^12^ Briefly, diffuse optics measures functional properties of deep tissue, i.e. tissue located several centimeters below the surface; the measurements are noninvasive and use nonionizing near-infrared radiation. These technologies, including diffuse optical spectroscopic imaging (DOSI), provide information about tissue optical absorption ($\mu_{a}$) and reduced optical scattering ($\mu_{s}^{\prime }$), from which concentrations of deoxygenated-hemoglobin (HHb) and oxygenated-hemoglobin (${\mathrm{HbO}}_{2}$), lipid, and water ($H_{2}O$) can be calculated. These quantities are then readily used to determine tissue total hemoglobin concentration (${\mathrm{Hb}}_{T}$) and oxygen saturation ($S_{t}O_{2}$).

Diffuse optics cannot replace x-ray mammography due to its limited spatial resolution.^12^^–^^14^ Nevertheless, DOSI and diffuse optical tomography (DOT) have demonstrated ability to locate lesions and to provide significant physiological contrast with respect to background tissue.^15^^–^^23^ Diffuse optics has also shown promise in distinguishing malignant from benign lesions,^15^^,^^18^^,^^20^^,^^23^^–^^28^ and therefore, it could play a role in the differential diagnosis of suspicious lesions. Finally, DOSI has also been demonstrated to successfully image tumors in patients with radiographically dense breasts^29^ for whom mammographic imaging is more challenging. Thus, diffuse optics could serve as a noninvasive adjunct imaging modality after lesion identification; the optical measurements could be performed and analyzed rapidly to reduce false positive rates, especially in young patients with dense breasts. If successful, diagnosis schemes with these supplemental optical biopsies could significantly reduce the number of lesions falsely identified as malignant by mammography and ultrasound, thereby eliminating some fraction of unnecessary invasive biopsies and reducing expense and patient stress. Furthermore, since diffuse optical techniques are cost effective, easily performed at the point-of-care, and free of ionizing radiation,^12^ the optical methodology could improve accessibility and be integrated in simple ways into the clinical standard-of-care.

Ideally, DOSI should identify the tumor region with respect to surrounding tissue and accurately classify lesions as malignant or benign; it should accomplish this goal even in the presence of substantial inter- and intrasubject tissue heterogeneity. To this end, the subject population presented herein (n=212) offers a unique opportunity. The subject population is large and includes both patients with malignant carcinomas of various subtypes and patients with benign lesions. All subjects were measured using the same DOSI technique, thereby providing consistency across the full sample. In contrast to prior work, the study utilized simple instrumentation that did not require other techniques for coregistration. We trained various logistic regression models on a subset of this patient population to differentiate malignant tissue from surrounding normal tissue, i.e., based on various DOSI-measured properties. The resultant models were then applied to a test set of patients with malignant tumors, benign lesions, and normal tissue.

The clinical study enabled critical examination of the ability of various models to locate lesions and perform differential diagnosis. Importantly, a z-score normalization and logistic regression technique^30^^–^^32^ was applied to the raw dataset and was found to render processed datasets that were more robust to inter- and intrapatient tissue heterogeneity. A recent publication^32^ demonstrated this methodology for prediction of which malignant tumors would achieve pathological complete response by the end of a neoadjuvant chemotherapy regimen. The present work measures all tissue types, but with a different output goal—to distinguish between malignant lesions and healthy tissue (or benign lesions). The present work also studied a much larger population compared to previous work, thereby permitting independent large training and test sets, rather than the small training set and leave-one-out protocol for testing of prior work; thus, the present study offers a rigorous test of all models/algorithms. We found that the z-score normalization and logistic regression technique significantly improved lesion classification and model calibration based on optically measured parameters. Specifically, the models using HHb, ${\mathrm{Hb}}_{T}$, and the tissue optical index $\left(\mathrm{TOI}=\frac{\mathrm{HHb}\cdot H_{2}O}{\text{Lipid}}\right)$ were shown to differentiate malignant tissue from both normal tissue and benign lesions with improved or similar accuracy compared to models with un-normalized or ratio-normalized data; additionally, Hosmer–Lemeshow analyses showed that the z-score-based models are well calibrated while the latter are not. Our results merit further testing in a larger population, but the size and heterogeneity of the current subject population, the statistical methods used, and the absence of required constraints derived from other imaging modalities are all factors that position this work as an important piece of evidence for the utility of diffuse optics in the breast cancer diagnostic setting.

## 2 Materials and Methods

### 2.1 Subjects

For this analysis, a database of n=212 subjects imaged with DOSI devices across eight different institutions (University of Pennsylvania; University of California, Irvine; University of California, San Francisco; Massachusetts General Hospital; Dartmouth Hitchcock Medical Center; Boston University; MD Anderson Cancer Center; and Dankook University) was utilized. Subjects provided written informed consent, and the HIPAA-compliant protocols and informed consent documents were approved by each site’s Institutional Review Board. The 212 subjects were women between the ages of 20 and 77 with breast lesions of at least 1 cm in length along the greatest dimension. Within this dataset, 181 subjects had biopsy-confirmed invasive ductal carcinoma (IDC), invasive lobular carcinoma (ILC), or both, and 31 subjects had a benign lesion. Table 1 contains demographic information for all subjects, as well as tumor histology, immunohistochemistry, and molecular subtype breakdowns for the subjects with malignant and benign lesions.

**Table 1:** Physiological malignant and benign lesion properties. Demographic, histological, and immunohistochemical data for all subjects in the dataset. The subject data are divided into malignant (n=181) and benign (n=31) lesion groups. For histological information, IDC refers to invasive ductal carcinoma, ILC refers to invasive lobular carcinoma, DCIS is ductal carcinoma *in-situ*, and LCIS is lobular carcinoma *in-situ*. ER, PR, and Her2 represent estrogen receptor, progesterone receptor, and human epidermal growth factor receptor status, respectively. HR positive refers to tumors that were hormone receptor positive but could not be classified as luminal A or luminal B due to unknown Ki-67.

	Malignant (n=181)	Benign (n=31)
Age, years		
Mean ± st. dev. (range)	50.2±11.8 (26 to 77)	40.5±11.9 (20 to 69)
Menopausal status, n (%)		
Pre-	86 (48%)	25 (81%)
Peri-	11 (6%)	1 (3%)
Post-	84 (46%)	5 (16%)
Maximum tumor size, mm		
Mean ± st. dev. (range)	34.6±21.7 (10 to 120)	19.5±7.8 (10 to 39)
Histological status, n (%)		
IDC	133 (73%)	—
ILC	9 (5%)	—
IDC + DCIS	28 (15%)	—
ILC + LCIS	2 (1%)	—
IDC + ILC	5 (3%)	—
Other malignant	4 (2%)	—
Fibroadenoma	—	20 (65%)
Cyst	—	5 (16%)
Other benign	—	6 (19%)
ER status, n (%)		
Positive	129 (71%)	—
Negative	49 (27%)	—
Unknown	3 (2%)	—
PR status, n (%)		
Positive	115 (64%)	—
Negative	63 (35%)	—
Unknown	3 (2%)	—
Her2 status, n (%)		
Positive	50 (28%)	—
Negative	121 (67%)	—
Equivocal	2 (1%)	—
Unknown	8 (4%)	—
Molecular subtype, n (%)		
Her2 positive	16 (9%)	—
HR positive	12 (7%)	—
Luminal A	36 (20%)	—
Luminal B	82 (45%)	—
Triple negative	27 (15%)	—
Unknown	8 (4%)	—

The subjects in this dataset were measured across a variety of imaging studies, and the present retrospective analysis was performed using the subset of subjects considered evaluable based on criteria relating to data quality and acquisition fidelity.^17^^,^^33^^–^^35^ Although some subjects were measured longitudinally, e.g., throughout the course of a chemotherapy regimen,^32^^,^^34^ only the pretherapy measurements are presented for analysis herein. Thus, the tissue has not been altered by chemotherapy or any other treatment regimen.

### 2.2 Optical Imaging Methods

The DOSI technique used in this study combines multispectral frequency-domain and broadband diffuse optical spectroscopy to measure tissue concentrations of oxygenated hemoglobin (${\mathrm{HbO}}_{2}$), deoxygenated hemoglobin (HHb), water ($H_{2}O$), and lipid; in addition, the tissue scattering amplitude (A) and power (b), as defined by a simplified Mie scattering model, where $\mu_{s}^{\prime }=A\lambda^{-b}$,^36^ are obtained. The combination of these measured parameters permits calculation of total tissue hemoglobin concentration (${\mathrm{Hb}}_{T}={\mathrm{HbO}}_{2}+\mathrm{HHb}$), tissue oxygen saturation ($S_{t}O_{2}={\mathrm{HbO}}_{2}/{\mathrm{Hb}}_{T}$), the tissue reduced scattering coefficient ($\mu_{s}^{\prime }$), and a tissue optical index $\left(\mathrm{TOI}=\frac{\mathrm{HHb}\cdot H_{2}O}{\text{Lipid}}\right)$. A more complete description of the DOSI method and instrument is given in Ref. 33. An American College of Radiology Imaging Network multicenter trial demonstrated the consistency and quality of multiple individual DOSI instruments across two years and seven measurement sites.^35^

**Fig. 1:**
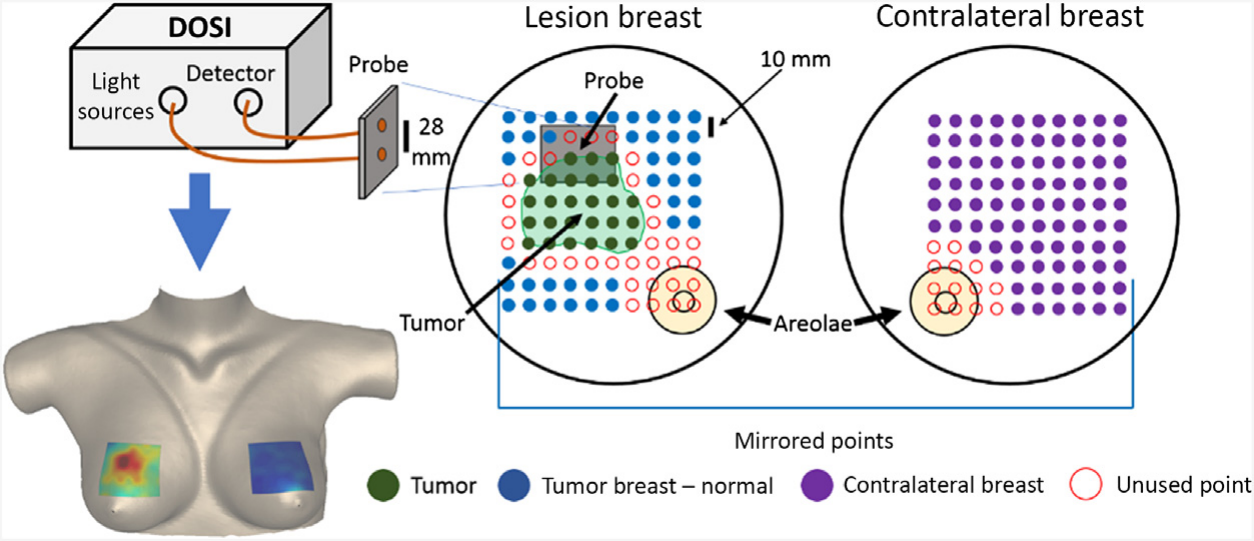
Schematic of DOSI measurement and region definition for differential diagnosis. Bottom left: A sample DOSI image projected onto a three-dimensional breast surface. Top left: DOSI instrument and probe. Right: A grid of points, over a surface area ranging from 7  cm×7  cm to 15  cm×16  cm, was measured on the lesion-bearing breast. This grid was chosen to encompass both the tumor and a portion of surrounding healthy tissue. The grid of points was marked using a transparency, which was then used to mirror the grid for measurements on the contralateral breast. The tumor region was chosen to be a region with a volume equal to the known tumor size, as measured with ultrasound, centered about the point of maximum TOI. The normal region of the tumor-bearing breast was defined as the set of points farthest away from the tumor region, excluding the areola. The contralateral breast normal region was defined as all measured points, excluding the areola.

DOSI measurements were made at a grid of distinct points on the tumor-bearing breast. This grid was chosen to encompass the entire tumor and surrounding normal tissue; it ranged in size from 7  cm×7  cm to 15  cm×16  cm, with an average size of approximately 10  cm×10  cm. The tumor location was determined via ultrasound and/or palpation. A mirrored grid of points was measured on the contralateral breast. The two measurement grids enabled definition of three distinct regions. The first region is tumor tissue; it was defined as the region of known dimensions and orientation of the tumor, as measured by ultrasound, centered about the point of maximum TOI. Notably, although TOI was used to center the lesion location, the definition of the tumor extent was independent of any optical parameters. The second region is the normal tissue on the tumor-bearing breast; it was defined as a set of points as far away from the tumor region as possible on the measurement grid, excluding the areola. This approach for defining the normal region helps to prevent any signal contamination from the tumor region due to the partial volume effect or uncertainty in the exact tumor boundary. The areola was excluded because of its intrinsic high blood flow and scattering, which is more similar to tumor tissue than normal tissue. Finally, the contralateral breast tissue, i.e., a third region which is comprised of normal tissue, was defined as the entire grid on the contralateral breast outside the areolar region. Figure 1 provides a schematic of these DOSI grid measurements and a sample image. At least 9 points per region were required to consider the subject analyzable in order to ensure the robustness of the mean value in each region.

### 2.3 Statistical and Analytic Methods

Since TOI has been empirically shown to distinguish malignant tissue from healthy tissue in individual subjects,^17^ significant interest exists in the community per determining the ability of TOI to distinguish between malignant lesions, benign lesions, and healthy tissue across a subject population. The goal of our analysis was to develop diagnostic metrics using TOI and other DOSI-measured parameters and then apply and evaluate the metrics across multiple tissue types.

The simplest diagnostic metric is an un-normalized value of a DOSI-measured parameter. For example, TOI has a range of values and cutoffs can be defined to optimally segregate tissues that are considered normal from those that are malignant. In practice, tumor-to-normal ratios of TOI (i.e., ${\mathrm{TOI}}_{T/N}$) improve upon the un-normalized TOI by accounting for some of the intersubject variability in the systemic levels of DOSI-measured physiological parameters. However, because healthy breast tissue also exhibits significant intrasubject heterogeneity in these quantities,^30^^,^^37^^,^^38^ a metric that takes into account the normal tissue heterogeneity might be expected to be more robust and to account more completely for heterogeneity in the problem. With this goal in mind, we constructed and utilized a z-score normalization scheme,^30^^–^^32^ which transforms the logarithm of a DOSI-measured parameter on the tumor-bearing breast to a z-score relative to the mean and standard deviation of the same parameter in the healthy tissue of the contralateral breast. This z-score parameter is defined as

$$Z_{j}=\frac{\ln\;X_{j}-\langle \ln\;X_{j_{\mathrm{Cont}}}\rangle }{\sigma [\ln\;X_{j_{\mathrm{Cont}}}]}.$$

Here, $X_{j}$ is the value of an un-normalized measured parameter j from a single spatial location on the tumor-bearing breast (e.g., the parameter j could be $S_{t}O_{2}$); $X_{j_{\mathrm{Cont}}}$ is the value of the un-normalized measured parameter j at a spatial point on the contralateral breast. $\left\langle \ln\;X_{j_{\mathrm{Cont}}}\right\rangle$ and $\sigma [\ln\;X_{j_{\mathrm{Cont}}}]$ represent the mean and standard deviation, respectively, over all points on the contralateral breast. $Z_{j}$ is thus the z-score for a given spatial measurement on the tumor-bearing breast relative to the healthy contralateral breast tissue for the j’th parameter. The $Z_{j}$ parameters are then separately averaged over all spatial points in the tumor region and in the normal region on the tumor-bearing breast, resulting in an average tumor $Z_{j}$ and an average normal $Z_{j}$ for each subject. Importantly, this z-score normalization also transforms the distribution of values of the data points to be approximately Gaussian and centered about $Z_{j}=0$; this feature improves the robustness of statistical algorithms such as logistic regression applied to these data. Notably, there were some small differences in the Z-score normalization technique between present and prior work,^32^ e.g., in the present work, the tumor and healthy ipsilateral tissue parameters were normalized to contralateral healthy tissue rather than ipsilateral healthy tissue.

The resultant tumor and normal $Z_{j}$ values can be used to run a logistic regression algorithm,^39^ which produces a model that optimally classifies each data point as either malignant or healthy based on the chosen parameter $Z_{j}$. Briefly, a malignancy parameter M is fit to maximize the likelihood estimation. For a single parameter model, M is given by

$$M^{i}=\beta_{o}+\beta_{j}\cdot Z_{j}^{i}.$$

Here, $M^{i}$ is the given model’s log odds of malignancy for the i’th subject; $\beta_{o}$ is the intercept term of the fitted weight vector; $\beta_{j}$ is the weighting term for the j’th measured parameter used in the model; $Z_{j}^{i}$ is the z-score for the j’th measured parameter of the i’th subject. For this analysis, the full weight vector $\overset{\to }{\beta }$ is then

$$\overset{\to }{\beta }=[\beta_{o},\beta_{j}].$$

A positive $\beta_{j}$ value indicates that higher values of the j’th parameter, relative to the normal tissue on the contralateral breast, are correlated with malignancy while a negative $\beta_{j}$ value indicates an inverse correlation with malignancy. The $\vec{\beta }$ weight vector is fit using MATLAB^®^’s native logistic regression function, mnrfit.^40^ The malignancy parameter M can then be transformed into a probability of malignancy, $P_{M}$, using a logistic function

$$P_{M}=\frac{1}{1+e^{-M}}.$$

The parameter $P_{M}$ represents the probability that a sampled tissue is malignant. It has a range from 0 to 1, and it can readily be used to predict the malignancy status of the tissue, depending on threshold levels. In this work, univariate models were developed for HHb, ${\mathrm{HbO}}_{2}$, ${\mathrm{Hb}}_{T}$, $S_{t}O_{2}$, lipid, $H_{2}O$, and TOI.

Once the probability of malignancy $P_{M}$ metric has been determined, it must be tested to analyze its discriminatory ability for malignant and nonmalignant tissue. This is achieved by applying the weight vector $\vec{\beta }$ to all tumor and normal regions across the subjects in the test set, i.e., subjects who were explicitly left out of the training set, and then calculating $P_{M}$ for these test data subjects. Importantly, this approach provides a validation of the fitted model that is not biased toward the sample on which the training was performed.^41^ The quality of the predictions, i.e., how well the predictions correspond to actual tissue type, is determined via receiver operating characteristic (ROC) analysis.^41^^–^^43^ For this particular ROC analysis, the area under the curve (AUC) values and their 95% confidence intervals were calculated using DeLong’s method.^44^

In addition to discriminatory ability, all models were also tested for their calibration, i.e., the degree to which the predicted probabilities of malignancy correspond to the actual rate of malignancy in the data. This analysis was performed using the Hosmer–Lemeshow method,^39^ a goodness-of-fit metric for classification models which subdivides the subject population by model-predicted probability of malignancy and compares the expected and actual probabilities within each group.

**Fig. 2:**
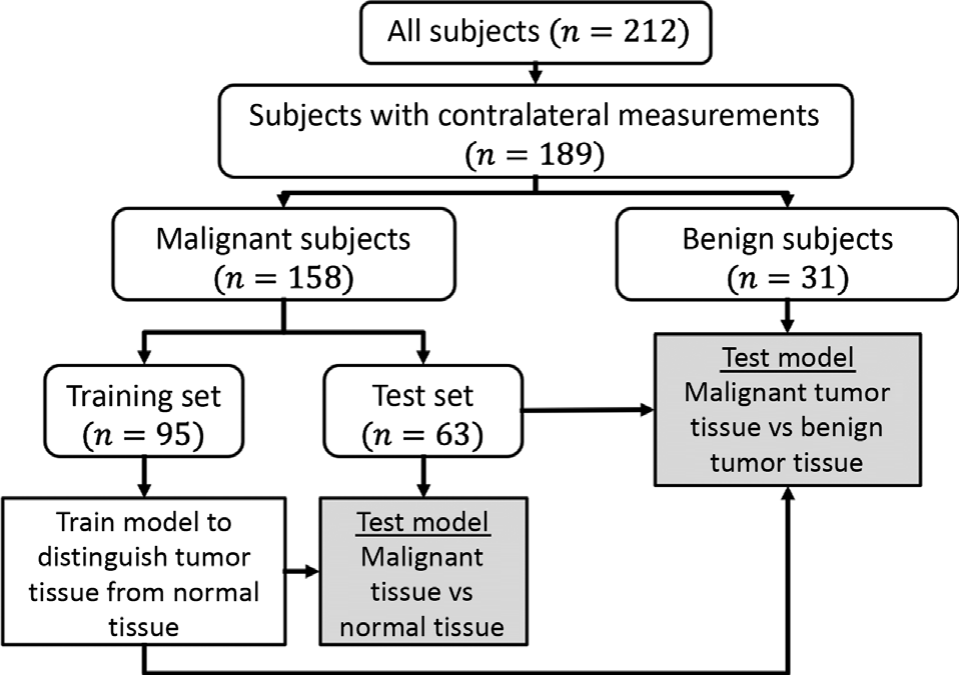
Flowchart of subject population division for differential diagnosis. The full dataset contains n=212 subjects; n=23 were excluded because they did not undergo contralateral breast measurements. Of the remaining 189 subjects, 158 had biopsy-confirmed malignant tumors and 31 had benign lesions. The subjects with malignant lesions were further subdivided into an n=95 subject training set and an n=63 subject test set. The training set was used to train logistic regression models to distinguish between malignant and normal tissue using various DOSI-measured parameters. These models were then applied to the tumor and healthy tissues in the test set to validate the models’ abilities to differentiate malignant lesions from surrounding normal tissue. The same models were then applied to the n=63 malignant tumors in the test set and the n=31 benign lesions to determine their abilities to categorize lesions as malignant or benign.

In the present work, the probability of malignancy model was trained using only the tumor and healthy tissue for subjects with biopsy-confirmed invasive carcinomas, and the z-score was normalized to the tissue in the contralateral breast. To enable this z-score normalization, the subjects (n=23) without contralateral breast measurements were excluded. Additionally, the subjects with biopsy-confirmed benign lesions (n=31) were not used to train the model; this approach maximized contrast between known healthy and malignant tissues. The remaining subjects with malignant lesions still comprised a large dataset. Therefore, a subset of these subjects was randomly selected and set aside to serve as an independent test set for the trained model. In practice, 60% of the subjects (n=95) were used to train the model and 40% (n=63) were set aside as a test set for independent validation. In addition, the fitted model was applied to the lesion and healthy tissues for the n=31 subjects that had benign masses. This additional application provides information about the degree to which benign lesions can be distinguished from malignant tumors using the same metric that differentiates malignant lesions from surrounding healthy tissue. Figure 2 contains a flowchart detailing the subdivision of the full dataset.

## 3 Results

Univariate logistic regression models aiming to differentiate malignant tissue from the surrounding normal tissue were run for all z-score normalized DOSI-measured parameters (HHb, ${\mathrm{HbO}}_{2}$, ${\mathrm{Hb}}_{T}$, $S_{t}O_{2}$, lipid, $H_{2}O$, and TOI) in the specified test set. These models were then applied to both the malignant tumors and healthy tissue in the test set and to the lesion tissues of subjects with benign tumors. Each model can thus be evaluated by two performance metrics: (1) ability to distinguish between malignant tumors and healthy tissue, which is what the model was trained to do, and (2) ability to distinguish malignant tumors from benign lesions.

**Fig. 3:**
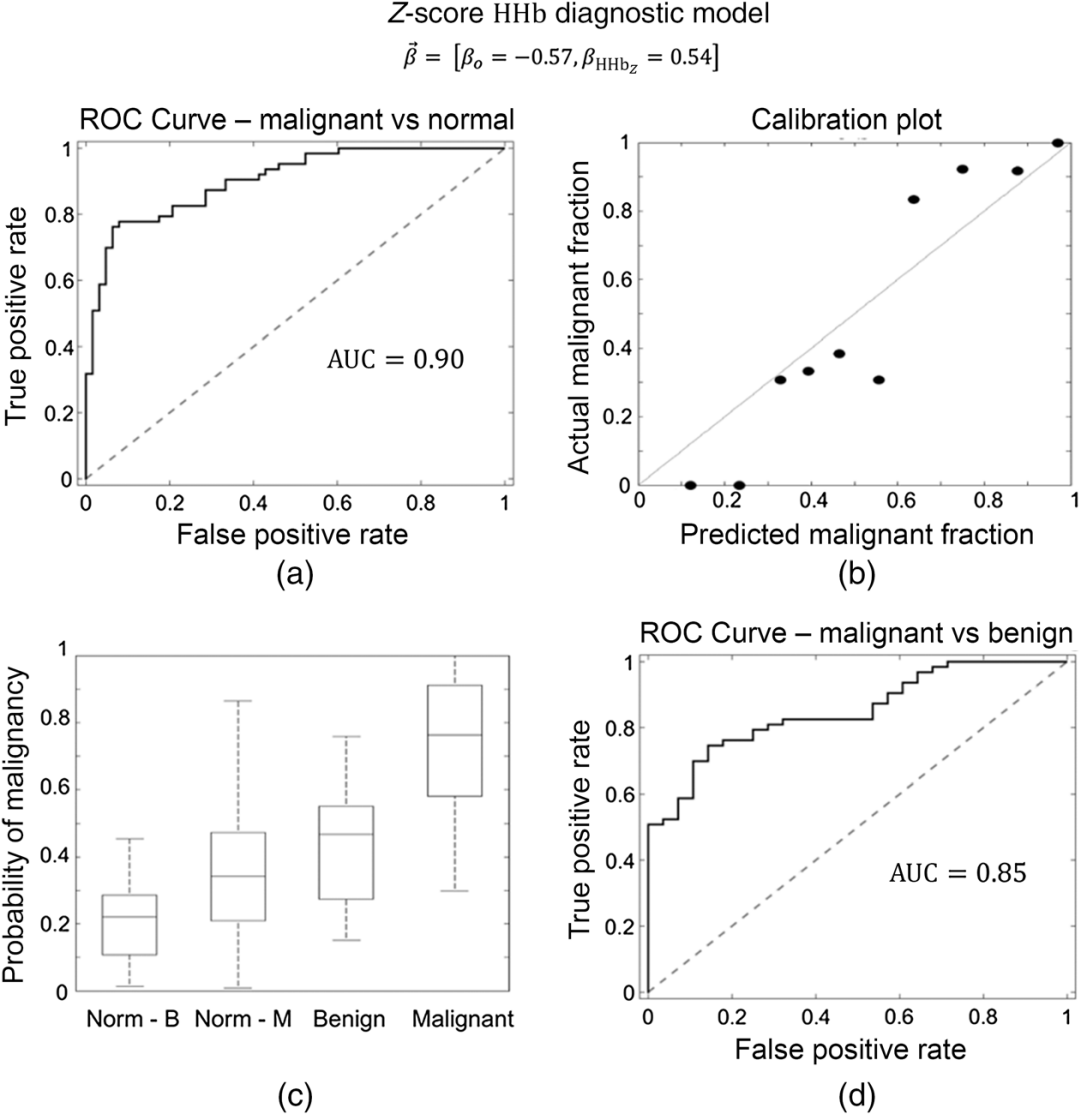
Z-score normalized deoxy-hemoglobin diagnostic model. (a) ROC curve for discrimination between malignant lesions and normal tissue. (b) Ten-group Hosmer–Lemeshow calibration plot comparing the actual fraction of tissue regions in the group that were malignant versus the predicted fraction of tissue regions in that group that should be malignant based on their individual probability of malignancy $P_{M}$ values. A well-calibrated model will have points that approximately lie along the identity line. (c) Boxplots of $P_{M}$ are divided into four groups: (1) Norm B: normal tissue for subjects with benign lesions, (2) Norm M: normal tissue for subjects with malignant tumors, (3) benign: benign lesions, and (4) malignant: malignant tumors. The hinges of the boxplots represent the first and third quartiles of the data and the whiskers represent the range of measurements within a distance 1.5× the interquartile range. It is noteworthy that the norm M and benign groups are significantly different with a p-value of 0.042, calculated via the independent t-test. Each other combination of the two groups is significantly different with a p-value of less than 0.001. (d) ROC curve for discrimination between malignant lesions and benign lesions. This model provides very good diagnostic ability for both malignant versus normal tissue and malignant versus benign lesions. The separation between these three tissue types can be seen in the $P_{M}$ boxplots where all three groups are distinguishable. The $\vec{\beta }$ weight vector for the model is given at the top of the figure.

HHb, TOI, and ${\mathrm{Hb}}_{T}$ proved to be the best parameters for predicting malignancy. The z-score normalized HHb model had an AUC=0.90 [95% Confidence Interval (CI): 0.85 to 0.95] for malignant versus normal tissue, and an AUC=0.85 (95% CI: 0.77 to 0.93) for malignant versus benign lesions (Fig. 3). The z-score normalized TOI model had an AUC=0.88 (95% CI: 0.82 to 0.94) for malignant versus normal tissue and AUC=0.85 (95% CI: 0.77 to 0.93) for malignant versus benign lesions. Z-score normalized ${\mathrm{Hb}}_{T}$ data produced malignant versus normal tissue predictions that were slightly worse than either HHb or TOI (AUC=0.83 (95% CI: 0.76 to 0.90); however, ${\mathrm{Hb}}_{T}$ produced similar malignant versus benign predictions (AUC=0.90 (95% CI: 0.84 to 0.96). These models indicate that higher values of HHb, TOI, or ${\mathrm{Hb}}_{T}$ were predictive of malignancy, which would indicate that malignant tumors have higher blood volumes than other breast tissues. These effects can be understood from the values of the $\overset{\to }{\beta }$ weight vectors (HHb in Fig. 3 and all models in Table S1 in the Supplementary Material). Notably, the ${\mathrm{HbO}}_{2}$, $H_{2}O$, and lipid concentrations were also predictive of malignancy; however, none of these parameters performed as well as HHb, TOI, or ${\mathrm{Hb}}_{T}$. The AUC values for all parameters can be found in Table S1 in the Supplementary Material.

**Fig. 4:**
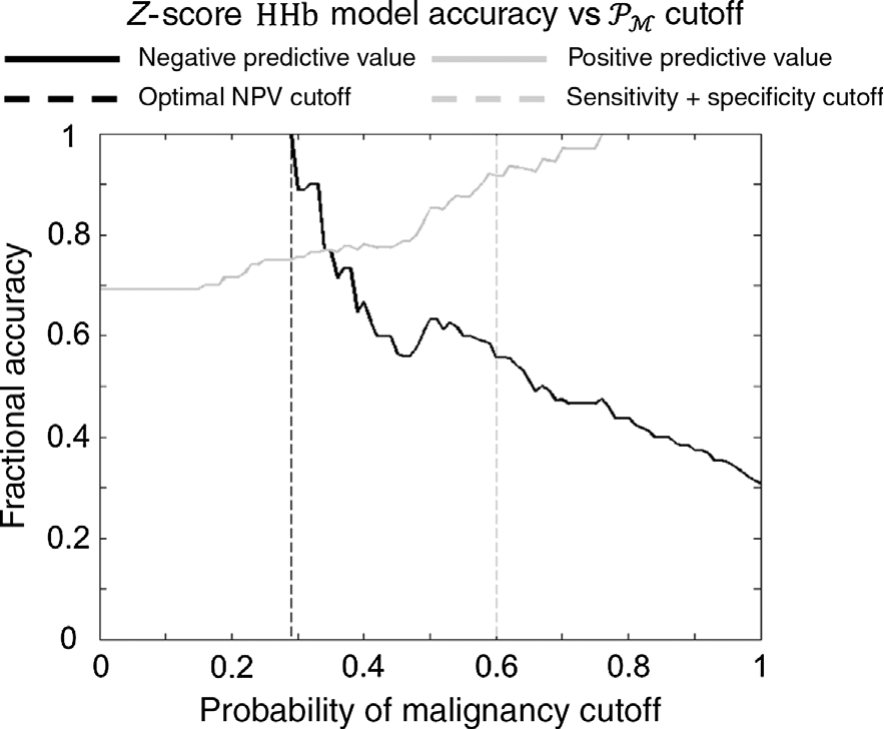
PPV and NPV versus probability cutoff——HHb model. The PPV, which increases as the probability of malignancy ($P_{M}$) cutoff increases, and the NPV, which decreases as the $P_{M}$ cutoff increases, are plotted versus $P_{M}$ cutoff. Choosing the $P_{M}$ cutoff to maximize the sum of sensitivity and specificity (gray dashed line) often provides a maximum overall classification accuracy. However, to confidently determine that all lesions predicted to be benign are actually benign, we would optimize the NPV with a lower cutoff value (black dashed line). Thus, these models can be tuned to optimally perform the chosen application.

The effect of the chosen cut-off values on the positive and negative predictive values (PPV and NPV, respectively) and the overall classification accuracy was also explored (see Fig. 4). For example, with the HHb model, if the cutoff was chosen to maximize the sum of the sensitivity and specificity, then the cutoff would be $P_{M}=0.59$, producing an accuracy of 77%, a PPV of 92%, and an NPV of 59%. However, if instead, we chose to maximize NPV (see Sec. 4 for more information), then, with a cutoff of $P_{M}=0.29$, the overall accuracy is 77%, the PPV is 75%, and the NPV is 100%.

To assess the benefits of the z-score normalization scheme, additional univariate logistic regression models were created using data that were un-normalized and using data that were ratio normalized to the tissue in the contralateral breast. The discriminatory ability, as assessed by the AUC, and calibration, as determined by the Hosmer–Lemeshow p-value, of these models using HHb, TOI, and ${\mathrm{Hb}}_{T}$ can be found in Table 2.

**Table 2:** Data normalization comparison—discriminatory ability and calibration. Univariate logistic regression models were created for all DOSI-measured parameters using three different types of data normalization: (1) no normalization, (2) tumor-to-normal ratio normalization, and (3) z-score normalization. Here, the models for HHb, ${\mathrm{Hb}}_{T}$, and TOI are shown. Models were evaluated on their ability to discriminate (1) malignant tumor tissue from the surrounding normal tissue and (2) malignant tumors from benign tumors. This discriminatory ability was assessed using the AUC of the ROC analysis for each model. The calibration of each model, i.e., the degree of agreement between the nominal probability of malignancy produced by the regression model and the actual probability of malignancy, was evaluated using the Hosmer–Lemeshow p-value. For the Hosmer–Lemeshow test, p<0.05 indicates that the model’s probabilities are not properly calibrated. Thus, well-calibrated models will have p>0.05. Z-score normalization and tumor-to-normal normalization both improve upon the discriminatory ability of un-normalized data models for differentiating malignant tumors from benign tumors. However, only z-score normalization produces good discrimination and well-calibrated probabilities for distinguishing malignant tumors from both the surrounding normal tissue and from benign tumors.

Model data	Malignant tumor versus healthy tissue		Malignant tumor versus benign lesion	
	AUC (95% CI)	Hosmer–Lemeshow p-value	AUC (95% CI)	Hosmer–Lemeshow p-value
Un-normalized				
HHb	0.85 (0.78 to 0.92)	0.056	0.64 (0.51 to 0.76)	0.000
${\mathrm{Hb}}_{T}$	0.78 (0.70 to 0.86)	0.614	0.65 (0.52 to 0.78)	0.016
TOI	0.80 (0.72 to 0.88)	0.000	0.53 (0.40 to 0.67)	0.000
Tumor-to-normal				
HHb	0.91 (0.85 to 0.96)	0.046	0.85 (0.78 to 0.93)	0.712
${\mathrm{Hb}}_{T}$	0.81 (0.74 to 0.89)	0.234	0.88 (0.81 to 0.95)	0.024
TOI	0.89 (0.84 to 0.95)	0.005	0.86 (0.79 to 0.94)	0.010
Z-score				
HHb	0.90 (0.85 to 0.95)	0.183	0.85 (0.77 to 0.93)	0.673
${\mathrm{Hb}}_{T}$	0.85 (0.76 to 0.90)	0.144	0.90 (0.84 to 0.96)	0.051
TOI	0.88 (0.82 to 0.94)	0.240	0.85 (0.77 to 0.93)	0.091

## 4 Discussion

The primary goal of this study was to analyze optical breast cancer data from a large and heterogeneous subject pool and evaluate whether z-score normalized DOSI metrics combined with logistic regression can accurately differentiate between malignant tumors, normal tissue, and benign lesions; a second goal was to critically examine the value of this methodology versus more traditional analyses applied to the same dataset. To this end, tumor and normal tissues from n=95 subjects with biopsy confirmed invasive carcinomas were used to train logistic regression models to predict malignancy. Each model was then applied to an independent test set of n=63 subjects with invasive carcinomas to assess the model’s ability to discriminate malignant from healthy tissue. The models were also applied to measurements of lesion tissue in n=31 subjects with benign masses to test for ability to distinguish malignant and benign masses.

Our findings represent the result of a robust statistical procedure utilizing a large database of diffuse optically measured breast tumors and using independent training and test validation datasets to minimize training bias. Importantly, each prediction model can be used to (1) localize lesions with respect to the surrounding normal tissue and (2) determine whether that lesion is malignant or benign. Ultimately, models such as these could be used for noninvasive optical biopsy or as a means of accurately identifying tumor and normal tissue to improve therapy monitoring.^32^

The three optically measured parameters that consistently performed the best were deoxy-hemoglobin concentration (HHb), total hemoglobin concentration (${\mathrm{Hb}}_{T}$), and the tissue optical index $\left(\mathrm{TOI}=\frac{\mathrm{HHb}\cdot H_{2}O}{\text{Lipid}}\right)$. The discriminatory ability of these features and the fitted β values from the logistic regression models indicate that higher blood volume and TOI is predictive of malignancy. Other parameters, such as ${\mathrm{HbO}}_{2}$, water, and lipid concentrations, were also able to discriminate malignant tumors from both normal tissue and benign lesions (Table S1 in the Supplementary Material). Additionally, normalization improves the ability of models to accurately predict malignancy (Table 2). Tumor-to-normal ratio normalized data and z-score normalized data both produce models with comparable discriminatory abilities, as measured by AUC, though z-score performed slightly better for most cases; both data types offer improvement over models using un-normalized data. Notably, models that used z-score normalized data also provided well-calibrated probabilities of malignancy, as measured by the Hosmer–Lemeshow test; the calibration of un-normalized data models and tumor-to-normal data models, by contrast, was generally poor. These findings suggest that simple un-normalized cut-off values may be insufficient to differentiate malignant and benign lesions. The findings also suggest that z-score normalization, which accounts for both inter- and intrasubject tissue heterogeneity, is the most beneficial data type for diffuse optical diagnosis in this dataset.

We also explored multifeature regression, but found that it did not significantly improve upon these single-parameter models. This lack of improvement could be a result of the high correlation between DOSI parameters or the heterogeneity of malignant lesions in this dataset. Other methodologies, such as k-fold validation (with k=3, 5, and 10) and support vector machine (SVM) learning, were explored, but the results they produced did not differ significantly from those presented here. Interestingly, while HHb, ${\mathrm{Hb}}_{T}$, and TOI produce similar discriminatory results, the use of HHb or ${\mathrm{Hb}}_{T}$ may have a logistical advantage with respect to TOI. This logistical advantage arises because most DOS or DOT systems use two to five distinct wavelengths, and more often than not, they only reconstruct HHb and ${\mathrm{HbO}}_{2}$ concentrations. Thus, if a model that relied only on HHb or ${\mathrm{Hb}}_{T}$ can produce predictions of equal quality to one that used TOI, then the HHb or ${\mathrm{Hb}}_{T}$ models might be preferable because they could be applied to measurements from a wider range of optical instrumentation. On the other hand, one must also consider that our DOSI system’s broadband reconstruction of the absorption and reduced scattering coefficients might constrain the measurements of HHb and ${\mathrm{HbO}}_{2}$ better than instruments that utilize only a few wavelengths of light; in this case, DOSI could provide more accurate measurements of these parameters.

In total, this work takes steps toward determining the optimal role for DOSI in the diagnostic setting. Though it is unlikely to replace gold-standard invasive biopsy as a means of determining malignancy, DOSI could be used as a preliminary screening tool to prevent clearly unnecessary biopsies for obviously benign lesions. For example, if the probability of malignancy cut-off value was set to be very low, i.e., relatively close to 0, then a very high negative predictive value could be achieved. In this case, only patients with clearly benign lesions would be identified as benign (see Fig. 4). These subjects could then avoid undergoing a costly, invasive biopsy that is extremely unlikely to yield a positive result. This scheme could be of particular use for subjects with high radiographic density breasts who are prone to false positives in x-ray mammography. The ideal cut-off and prediction metric for this type of screening would require further validation, but could ultimately save patients time, reduce expense, and reduce undue stress.

Several areas need to be explored further with respect to the diagnostic markers from this dataset. First, this was a retrospective analysis performed with knowledge of the malignant or benign state of each tumor. A prospective, well-controlled study of suitable statistical power should be designed and performed to further validate the ability of the HHb, ${\mathrm{Hb}}_{T}$, and/or TOI z-score models we have developed and demonstrated. Additionally, in the present investigation, differentiation of malignant and benign lesions was determined using a prediction model that was trained to distinguish malignant from normal tissue, rather than benign lesions. It would be instructive to explore direct binomial logistic regression between invasive carcinomas and benign masses. If this analysis produced similar physiological correlations between HHb, ${\mathrm{Hb}}_{T}$, or TOI and malignancy, it would be further evidence of the robustness of these methods. Notably, this suggested approach was not attempted for the current dataset because of the approximately 5:1 disparity between the number of available malignant and benign lesions; such a discrepancy between the two training classes can significantly bias the fitting algorithm toward the larger class.^45^ Out dataset is also very heterogeneous (see Table 1). It would be beneficial to explore variations in optically measured parameters across tumor and patient characteristics and molecular subtype. We performed an initial investigation of these variations but observed no significant difference in HHb, ${\mathrm{Hb}}_{T}$, or TOI across any of the parameters presented in Table 1. For this reason, we believe that our models are robust across all breast tumor types. However, we plan to continue this investigation to make models more robust, for example, by optimizing model parameters and by exploring different classification schemes such as SVM and other machine learning algorithms. In a different vein, we will explore the characteristic optical properties of each tumor type which could enable improved normalization and lesion identification. Finally, we note that the models presented here utilized only optically measured tissue properties in the prediction models. The creation of models using a combination of DOSI-measured parameters and other mammographic or sonographic signatures could further improve classification ability and prevent unnecessary biopsies.^25^

## 5 Conclusion

A dataset of n=212 subjects, including those with malignant and benign lesions, were measured using DOSI. Logistic regression models utilizing single z-score normalized DOSI-measured parameters, and more traditional DOSI parameters, were created to distinguish malignant tissue from normal tissue in a subset of the subjects with malignant carcinomas. These models were then applied to an independent set of subjects with malignant lesions, and to all subjects with benign lesions, in order to test the model’s ability to discriminate both malignant from normal tissue and malignant from benign lesions. The best models used the deoxy-hemoglobin concentration (HHb), total hemoglobin concentration (${\mathrm{Hb}}_{T}$), or the tissue optical index (TOI) parameters. These models discriminated malignant tissue from normal tissue with AUCs of 0.90 (95% CI: 0.85 to 0.95), 0.83 (95% CI: 0.76 to 0.90), and 0.88 (95% CI: 0.82 to 0.94) for HHb, ${\mathrm{Hb}}_{T}$, and TOI, respectively. Interestingly, the same models for HHb, ${\mathrm{Hb}}_{T}$, and TOI could accurately distinguish malignant from benign lesions with an AUC=0.85 (95% CI: 0.77 to 0.93), 0.90 (95% CI: 0.84 to 0.96), and 0.85 (95% CI: 0.77 to 0.93), respectively. The results indicate that DOSI not only has the ability to distinguish malignancies from healthy tissue in a single subject, but also to differentiate between malignant and benign lesions within the same quantitative models. Notably, z-score normalization of the data produced metrics with better or similar predictive abilities relative to both un-normalized and ratio-normalized data (based on AUC); moreover, the z-score normalization regression was better calibrated than either un-normalized or ratio-normalized data (based on Hosmer–Lemeshow p-values).These findings, along with the inherent logistical advantages of diffuse optics, position DOSI as an attractive modality for performing preliminary, noninvasive biopsies, as a means of improving the conventional imaging paradigm for differential breast cancer diagnosis.

## Code, Data, and Materials Availability

Associated code and data can be made available upon request.
