# Supplementary material for: Breast cancer differential diagnosis using diffuse optical spectroscopic imaging and regression with z-score normalized data
Source: J Biomed Opt. 2021 Feb 23;26(2):026004. doi: 10.1117/1.JBO.26.2.026004 (PMC7901858; doi:10.1117/1.JBO.26.2.026004)
Supplement: Supplementary file 1 [file JBO_026_026004_SD001.pdf]

Supplementary Information

Parameter	β_o	β_i	AUC (95% CI): Malignant vs Healthy	AUC (95% CI): Malignant vs Benign
TOI	-0.63	0.34	0.88 (0.82-0.94)	0.85 (0.77-0.83)
HHb	-0.57	0.54	0.90 (0.85-0.95)	0.85 (0.77-0.93)
HbO_2	-0.40	0.36	0.75 (0.66-0.84)	0.85 (0.78-0.93)
Hb_T	-0.48	0.39	0.83 (0.76-0.90)	0.90 (0.84-0.96)
H_2O	-0.47	0.22	0.80 (0.72-0.88)	0.80 (0.70-0.90)
$Lipid$	-0.28	-0.19	0.72 (0.63-0.81)	0.65 (0.52-0.77)
S_tO_2	-0.05	-0.12	0.71 (0.62-0.80)	0.49 (0.36-0.61)

Supplementary Table 1. DOSI-Derived Prediction Models. Univariate malignancy prediction models were produced for all z-score normalized DOSI parameters. β_o and β_i are the intercept term and the parameter weighting term, respectively, for the given logistic regression model. Positive β_i values indicate that higher values of these quantities, relative to the normal tissue on the contralateral breast, are indicative of malignancy while negative values indicate an inverse correlation with malignancy. The AUC values and 95% confidence intervals, both for differentiating malignant lesions from normal tissue and malignant lesions from benign lesions, are also given.
